# Supplementary material for: Geometric spin echo under zero field
Source: Nat Commun. 2016 May 19;7:11668. doi: 10.1038/ncomms11668 (PMC4874036; doi:10.1038/ncomms11668)
Supplement: Supplementary Information — Supplementary Figures 1-3 [file ncomms11668-s1.pdf]

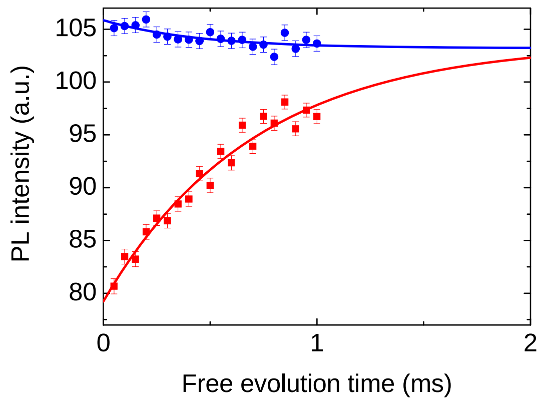


**Supplementary Figure 1 | The electron spin relaxation.** Time dependence of the pulsed ODMR signal prepared in the bright state $|B\rangle$ (red sqares) and the ancillary state $|0\rangle$ (blue circles). Solid lines show exponential decay fittings. The decay rate corresponded to the population decay time $T_1$ of 700 μs used in the fitting in Fig. 3c. Error bars are defined as the s.d. of the photon shot noise.

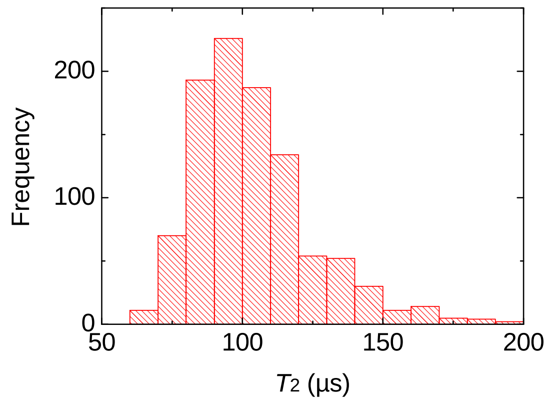


**Supplementary Figure 2 | Histogram of dimer $T_2$ under a zero magnetic field.** Theoretical distribution of the $T_2$ time under random dimer $^{13}C$ nuclear spin bath configuration, where we only consider the dimers within 4 nm of electron spin. The bath configuration giving $T_2$ = 75 μs is used for the calculation of Fig. 3c and 4a.

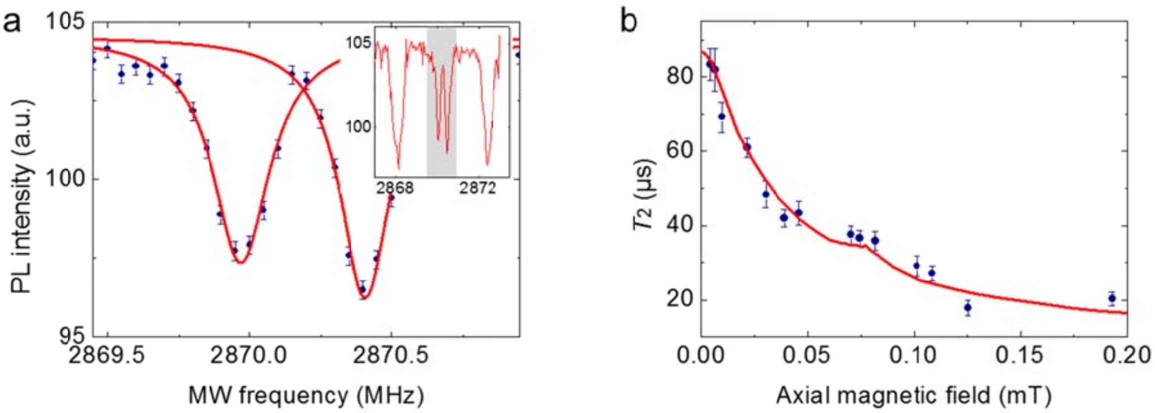


**Supplementary Figure 3 | Transverse zero-field splitting effect.** (**a**) Frequency dependence of the pulsed ODMR signal showing the strain splitting in the dip corresponding to the nuclear spin $m_\mathrm{I} = 0$ state. Solid lines are Lorenzian curve fittings. Inset shows the full spectrum of the pulsed ODMR, where the center part (grey area) corresponds to the $m_\mathrm{I} = 0$ state. (**b**) Echo coherence decay time $T_2$ as a function of magnetic field measured along the NV axis with considering the strain splitting of 0.23 MHz and inhomogeneous broadening of 0.43 MHz due to the hyperfine field from the spin bath. Error bars in (**a**) are defined as the s.d. of the photon shot noise. Error bars in (**b**) are defined as the s.d. of least squares fitting.
